# Financing Regimes and Case-Mix Complexity in Psychiatric Hospitals Beyond the Pandemic Shock—Insights from a Regional European Healthcare System

**DOI:** 10.3390/healthcare14091181

**Published:** 2026-04-28

**Authors:** Andrian Țîbîrnă, Floris Petru Iliuta, Mihnea Costin Manea, Mirela Manea

**Affiliations:** 1 Department of Psychiatry and Psychology, Faculty of Stomatology, “Carol Davila” University of Medicine and Pharmacy, 020021 Bucharest, Romania; 2 “Prof. Dr. Alexandru Obregia” Clinical Hospital of Psychiatry, 041914 Bucharest, Romania

**Keywords:** mental health services, case-mix complexity, health system resilience, financing structures, pandemic shock, regional disparities

## Abstract

**Background/Objectives**: The COVID-19 pandemic intensified concerns regarding the resilience and financing architecture of mental health services, yet it remains unclear whether crisis-induced adjustments fundamentally altered hospital case-mix complexity or merely exposed pre-existing structural configurations. This study examines the relationship between financing regimes and case-mix complexity in psychiatric hospitals in Romania, a Central and Eastern European health system characterized by mixed financing arrangements and pronounced interregional heterogeneity. **Methods**: Using administrative data comprising 752 hospital section–year observations (2019–2024), we identify structural financing–organization regimes through a two-step clustering procedure (hierarchical Ward method followed by K-means refinement) based on revenue composition, expenditure allocation, workforce structure, and operational pressure indicators. **Results**: Three distinct regimes emerge, reflecting persistent institutional configurations rather than temporary crisis-induced groupings. Chi-square tests confirm that regime membership is statistically independent of pandemic timing. A multivariate regression model controlling for financing composition and expenditure structure shows that structural variables (particularly the share of contract-based revenues and the allocation of expenditures) exert systematic and economically meaningful effects on the case-mix index (*CMI*). Pandemic and post-pandemic indicators do not retain robust explanatory power once structural determinants are accounted for. Regional robustness analyses further demonstrate that financing architecture consistently outweighs temporal shock effects in explaining territorial variation in clinical complexity. **Conclusions**: The findings suggest that psychiatric hospital case-mix dynamics are structurally embedded within differentiated financing regimes whose influence persists beyond crisis periods. By integrating regime identification with outcome modeling in a Central and Eastern European context, this study contributes to the international literature on health system resilience and highlights the primacy of institutional financing architecture over episodic shock effects in shaping hospital complexity.

## 1. Introduction

The COVID-19 pandemic generated unprecedented pressures on health systems worldwide [1,2,3], exposing structural vulnerabilities in financing arrangements, resource allocation mechanisms, and service delivery models. Mental health services were particularly affected, as psychiatric hospitals faced simultaneous increases in clinical complexity, disruptions in referral pathways, workforce shortages, and reallocation of financial resources [4,5,6]. While many studies in the international literature [7,8,9] have focused on the immediate operational consequences of the pandemic, considerably less attention has been paid to the question of whether pandemic-related shocks have fundamentally altered the dynamics of hospital cases or merely amplified pre-existing structural configurations.

In European health systems characterized by mixed financing arrangements, including social health insurance, public subsidies, and budgetary transfers, the pandemic prompted rapid adjustments in funding flows and expenditure priorities [10,11,12]. Temporary increases in emergency allocations, shifts toward personnel expenditures, and constraints on capital investment were frequently observed. However, an open empirical question remains: *did these pandemic-induced financing adjustments lead to structural transformations in hospital complexity, or did entrenched financing regimes continue to shape institutional performance beyond the crisis period?*

This study is theoretically grounded in a dual framework combining institutional theory and health system resilience. Institutional theory suggests that hospital behavior is shaped by stable incentive structures embedded in financing arrangements, contractual rules, and administrative routines, which tend to persist over time despite external shocks. In parallel, resilience theory distinguishes between temporary adaptive responses and deeper structural transformation, emphasizing that health systems may absorb shocks without fundamentally altering their institutional architecture. Within this perspective, financing regimes are conceptualized as latent structural configurations that mediate the relationship between exogenous shocks and observed performance outcomes, including case-mix complexity.

Hospital case-mix complexity, commonly operationalized through the case-mix index (*CMI*) within DRG-based systems, represents a key proxy for clinical intensity, resource consumption, and service differentiation [13,14]. Changes in the *CMI* may reflect shifts in patient severity, access patterns, treatment intensity, or institutional specialization. Yet disentangling pandemic effects from structural determinants is methodologically challenging, particularly in health systems with pronounced regional heterogeneity.

The Central and Eastern European context remains underexplored in this regard. Empirical evidence based on administrative hospital-level data from this region is limited, especially in the field of psychiatric services. Romania provides a particularly relevant setting, as its mental health system combines contract-based financing through the national health insurance fund with public subsidies and regionally differentiated expenditure structures [15,16,17]. This institutional configuration creates potential for persistent structural heterogeneity in hospital organization and financing regimes. Against this background, the present study investigates whether pandemic and post-pandemic shifts significantly altered case-mix complexity in Romanian psychiatric hospitals or whether structural financing regimes represent the dominant explanatory mechanism.

In this context, Romania represents a particularly relevant case for examining the interaction between structural financing arrangements and crisis dynamics. The coexistence of contract-based reimbursement mechanisms with public subsidies and regionally differentiated resource allocation creates a layered institutional environment in which hospitals may respond heterogeneously to external shocks. This makes it possible to empirically distinguish between temporary pandemic effects and deeper structural determinants of hospital performance.

The main aim of this study is to examine the relationship between financing structures and case-mix complexity in psychiatric hospitals in Romania, with specific attention paid to pandemic and post-pandemic periods. To achieve this aim, the study pursues the following objectives:

O.1. To identify structural typologies of psychiatric hospital financing and operational organization using multivariate clustering techniques.
O.2. To assess the temporal robustness of identified financing regimes across pre-pandemic, pandemic, and post-pandemic periods.
O.3. To evaluate the extent to which pandemic (PAN) and post-pandemic (POST) periods influence case-mix complexity.
O.4. To determine whether structural financing composition exerts a stronger effect on *CMI* than pandemic timing.
O.5. To explore regional disparities in case-mix complexity within identified financing regimes.

The analysis addresses the following research questions:

RQ.1.: Do distinct structural financing regimes characterize psychiatric hospitals in Romania?

RQ.2.: Are these regimes temporally stable across pandemic and post-pandemic periods?

RQ.3.: Does pandemic timing significantly affect hospital case-mix complexity once structural determinants are controlled for?

RQ.4.: To what extent do financing composition and expenditure structure explain regional disparities in case-mix outcomes?

Beyond its empirical contribution, this study has direct relevance for health policy and system design. By clarifying whether case-mix complexity is primarily driven by structural financing configurations or by temporary crisis effects, the analysis provides evidence on the effectiveness of existing reimbursement mechanisms and resource allocation strategies. In the Romanian context, where mental health services face persistent capacity constraints and regional disparities, understanding the structural drivers of hospital complexity is essential for designing targeted financing reforms, improving allocative efficiency, and ensuring the sustainability of psychiatric care provision.

The contribution of this study is threefold. It advances the literature on health system resilience by shifting the analytical focus from short-term pandemic shocks to structurally embedded financing regimes as primary determinants of hospital case-mix complexity. The study provides rare empirical evidence from a Central and Eastern European context, using administrative hospital-level data from Romania to enrich comparative research beyond Western European and OECD systems. Also, it adopts an integrated methodological approach that combines multivariate cluster analysis with regression-based outcome modeling, thereby linking latent institutional typologies to observable clinical complexity indicators and offering a more structurally grounded interpretation of hospital performance.

## 2. Literature Review

The analysis of the relationship between funding structure and hospital clinical complexity is part of a broad body of literature that intersects health economics, institutional theory, and studies on the resilience of public systems. Over the past two decades, research has progressively moved away from purely volume-based or cost-centric explanations of hospital performance and has begun to privilege the perspective of the institutional architecture of financing as a structural determinant of organizational behavior [18,19,20]. This conceptual shift reflects the recognition that hospitals do not respond exclusively to the absolute level of available resources, but rather to the configuration of incentives embedded in reimbursement mechanisms and budgetary structure [21,22,23].

In European systems characterized by mixed financing, combining contracts with social insurance funds, budget subsidies, and public transfers, the actual distribution of revenue sources and spending priorities generates differentiated organizational configurations at the institutional level. Even within a unified regulatory framework, hospitals can develop distinct profiles depending on the degree of contractual integration, the share of allocations for staff or investments, and the operational pressures associated with case volume. The institutional literature suggests that these configurations tend to become relatively stable over time, crystallizing into persistent organizational regimes anchored in administrative routines and resource allocation mechanisms that are difficult to reconfigure in the short term [24,25].

At the same time, the COVID-19 pandemic has generated a wave of research focused on the resilience of health systems, their ability to adapt to exogenous shocks, and the temporary effects of resource redistribution [26,27,28]. However, most studies have analyzed the immediate impact of the crisis on service volume, costs, or access to care, without systematically assessing whether the pandemic shock has structurally altered the relationship between funding architecture and clinical complexity [29,30,31,32]. Thus, it remains unclear whether the variations observed in performance indicators reflect profound institutional transformations or merely cyclical adjustments superimposed on pre-existing structural configurations.

This issue is particularly relevant in the field of mental health services. Psychiatric hospitals operate at the intersection of acute and chronic care, between admission pressure and budget constraints, and the complexity of the cases treated is influenced by both clinical severity and the structure of financial incentives [33,34,35]. The Case-Mix Index (*CMI*), widely used in DRG-based systems, thus becomes not only an indicator of clinical intensity, but also a result of the interaction between reimbursement mechanisms and organizational capacity [36,37,38].

In Central and Eastern European systems, this relationship has not been sufficiently investigated empirically. Most analyses of hospital financing and case complexity come from Western European systems or OECD economies with extensive statistical infrastructure [39,40,41]. In contrast, systems characterized by institutional transition, regional heterogeneity, and variable combinations of contractual financing and public subsidies remain relatively underrepresented in quantitative literature based on administrative panel data [42,43,44].

Therefore, this literature review aims to theoretically ground the analysis of the relationship between structural financing regimes and case complexity in psychiatric hospitals, clarify the debate on institutional stability in the face of pandemic shocks, and identify gaps in international research. The following sections analyze, in turn, the contributions regarding hospital funding typologies, the stability of institutional regimes in crisis contexts, the link between funding composition and clinical complexity, and the tension between structural determinants and the temporal effects of the pandemic.

### 2.1. Structural Financing Regimes in Psychiatric Hospitals

Health systems characterized by mixed financing arrangements frequently generate differentiated institutional configurations at the hospital level, even when they operate within a formally unified regulatory and reimbursement framework [45,46,47]. Variations in the relative weight of contract-based revenues, earmarked public subsidies, own-source revenues, and intergovernmental transfers tend to shape distinct financial profiles across institutions [48,49,50]. These differences are not merely accounting artifacts; rather, they structure managerial incentives, influence resource allocation priorities, and affect the strategic positioning of hospitals within the broader health system. Over time, recurring patterns in revenue composition, expenditure structure, workforce configuration, and operational workload may crystallize into relatively stable financing regimes. Such regimes reflect institutional path dependency and embedded resource allocation logics, rather than temporary managerial adjustments or short-term fluctuations in service demand [51,52].

The concept of a financing regime goes beyond the simple identification of dominant revenue sources. It implies the existence of internally coherent configurations in which financing structure, expenditure priorities, and organizational characteristics are mutually reinforcing. In hospital settings, a high reliance on contract-based revenues derived from social health insurance funds typically entails stronger exposure to case-based reimbursement incentives, greater emphasis on DRG coding accuracy, and closer alignment between clinical activity and contractual performance indicators [53,54,55]. Conversely, institutions more dependent on fixed public subsidies or budgetary transfers may exhibit different strategic behaviors, including greater stability of staffing structures, less responsiveness to marginal changes in case-mix intensity, or alternative service orientations [56,57]. These structural configurations tend to be reproduced over time through administrative routines, contractual renegotiations, and cumulative investment decisions, thereby reinforcing institutional heterogeneity within the same national system.

In psychiatric services, these dynamics become particularly salient. Unlike many acute care specialties characterized by short treatment cycles and highly standardized procedures, psychiatric hospitals operate at the intersection of acute episodes, chronic care management, rehabilitation services, and long-term social support [33,58,59]. The interaction between care intensity, workforce composition, and funding mechanisms is therefore especially tight. Personnel expenditures often represent a dominant share of total costs, given the labor-intensive nature of psychiatric treatment, while capital investments may be limited yet strategically important for specialized units [60,61,62]. Moreover, case-mix complexity in psychiatric settings is shaped not only by patient severity but also by referral patterns, length of stay practices, and institutional specialization. As a result, differences in financing composition are likely to translate into differentiated organizational profiles with implications for both clinical complexity and operational pressure [63,64].

Institutional theory provides an important analytical lens for understanding the persistence of such differentiated configurations. Healthcare organizations operate within dense regulatory environments that constrain rapid structural change [65,66]. Budgetary rules, contract templates, staffing norms, and reporting requirements create a framework of incentives and constraints that stabilizes financing patterns over time [67,68,69]. Even in the face of exogenous shocks or policy adjustments, deeply embedded financing architectures tend to display inertia. This path dependency implies that hospitals with historically high levels of contract-based integration or subsidy dependence are likely to maintain those structural characteristics unless confronted with substantial systemic reform [70,71,72]. Consequently, heterogeneity across hospitals should not be interpreted as random dispersion, but rather as the manifestation of underlying financing regimes.

Empirical studies in health economics have increasingly recognized that hospital performance cannot be fully explained by isolated variables such as total revenue or expenditure levels. Instead, combinations of financial and organizational indicators often cluster together in systematic ways [43,48,73]. Multivariate classification approaches applied in different national contexts have revealed recurring typologies of hospitals distinguished by their revenue mix, cost allocation patterns, staffing intensity, and workload ratios [74,75]. These findings suggest that the identification of regime-based configurations offers a more nuanced understanding of institutional diversity than single-variable analyses.

Within this theoretical and empirical framework, the identification of structural financing regimes in psychiatric hospitals constitutes a necessary first step in understanding performance dynamics [76,77,78,79]. If hospitals can indeed be grouped into internally coherent clusters defined by distinct financing and organizational profiles, then subsequent analysis of clinical complexity and cost patterns must account for these regime effects [19,80,81,82]. The existence of such regimes would imply that institutional heterogeneity is structured and systematic, rather than idiosyncratic. Accordingly, the following hypothesis is formulated:

**H1.**  *Psychiatric hospitals are characterized by distinct structural financing regimes reflecting heterogeneous institutional configurations.*

This hypothesis rests on three interrelated arguments. First, mixed financing arrangements generate variability in revenue composition and expenditure priorities. Second, the labor-intensive and clinically heterogeneous nature of psychiatric services amplifies the organizational implications of these financial differences. Third, institutional path dependency fosters the persistence of coherent configurations over time. If supported empirically, H1 would establish the foundational premise that psychiatric hospitals operate within differentiated structural regimes, thereby providing the conceptual basis for examining how these regimes shape case-mix complexity and performance outcomes in subsequent analyses.

### 2.2. Temporal Stability Beyond the Pandemic Shock

The COVID-19 pandemic generated an unprecedented systemic stress test for health systems worldwide. Across Europe, emergency reallocations of financial resources, temporary subsidies, revised reimbursement rules, and rapid organizational adjustments were introduced to preserve service continuity [30,31,83]. In many countries, hospital budgets were supplemented through extraordinary transfers, elective activity was suspended, and contractual arrangements were temporarily relaxed or recalibrated [84,85,86]. These interventions were frequently interpreted as signals of structural transformation. However, the presence of short-term financial and operational adjustments does not necessarily imply a redefinition of the underlying institutional architecture that governs hospital behavior.

From an institutional theory perspective, organizations embedded in highly regulated environments, such as public health systems, tend to exhibit strong structural inertia [87,88,89]. North’s theory [90] of institutional path dependency emphasizes that formal rules, contractual arrangements, and administrative routines create self-reinforcing trajectories that resist abrupt change. Similarly, neo-institutional frameworks highlight the stabilizing effect of regulatory isomorphism and bureaucratic continuity, particularly in sectors characterized by public accountability and centralized oversight [91,92]. Financing architectures, once embedded in legal frameworks and contractual templates, typically evolve incrementally rather than through abrupt regime shifts.

Empirical evidence emerging from the pandemic period supports this interpretation. Comparative analyses across European health systems have shown that while emergency funds temporarily altered revenue flows, core reimbursement mechanisms, especially DRG-based or contract-based financing structures, remained largely intact [86,93,94]. Studies examining hospital payment systems in Germany, France, and Italy indicate that extraordinary pandemic compensations were layered onto pre-existing structures rather than replacing them [95,96,97,98]. In most cases, DRG-based reimbursement formulas were temporarily supplemented by lump-sum compensations for capacity retention or revenue shortfalls, but the fundamental logic of case-based contracting persisted [99,100,101].

In addition, research on health system resilience has increasingly distinguished between adaptive flexibility and structural transformation. Various academic studies [102,103,104] conceptualize resilience not as systemic redesign, but as the ability to absorb shocks while maintaining core institutional functions. This perspective implies that temporary operational disruptions do not automatically translate into durable institutional reconfiguration. Indeed, analyses of post-pandemic hospital financing patterns in several OECD countries reveal that once emergency measures expired, revenue composition and expenditure structures reverted closely to pre-pandemic distributions [105,106,107].

Importantly, the persistence of financing regimes extends beyond European health systems and is widely documented across diverse international contexts. Evidence from the United States shows that hospital responses to systemic shocks remain strongly shaped by pre-existing reimbursement frameworks, particularly within prospective payment and DRG-based systems, where core incentive structures remain stable despite temporary policy interventions [108,109]. Similarly, studies from Asia and Latin America indicate that hybrid financing arrangements generate heterogeneous yet structurally persistent patterns of hospital behavior, even in settings characterized by ongoing reforms or fiscal pressures [31,76,110].

Across these contexts, financing architectures tend to exhibit strong path dependency, with hospitals adapting to shocks within established institutional and regulatory constraints rather than undergoing fundamental structural change. This cross-regional evidence aligns with institutional perspectives emphasizing the durability of incentive structures and the incremental nature of organizational adaptation.

Taken together, these findings suggest that the resilience of financing regimes reflects a broader institutional regularity, reinforcing the generalizability of the structural perspective adopted in this study and highlighting the dominant role of enduring financing configurations in shaping hospital performance and case-mix complexity [111,112].

In psychiatric services, structural inertia may be even more pronounced. Unlike high-technology acute specialties, psychiatric hospitals rely heavily on stable workforce configurations and long-term care pathways [33,113,114]. Staffing structures, bed allocations, and institutional specialization cannot be rapidly reengineered in response to short-term shocks. Studies focusing on mental health services during COVID-19 report temporary changes in admission patterns and outpatient activity but do not document systemic shifts in financing architecture [115,116,117]. In many countries, funding for psychiatric units was protected or stabilized to ensure continuity of care, reinforcing rather than disrupting pre-existing structural arrangements [118,119,120].

The concept of regime stability also finds support in organizational ecology theory, which posits that organizations adapt within constrained niches defined by regulatory and resource environments [121,122]. Hospitals embedded in specific financing configurations, such as high contract-based revenue dependence or strong reliance on subsidies, are unlikely to alter their structural profile solely due to a transient external shock [55,123,124]. Instead, adaptation occurs within the boundaries of the existing regime. Even when temporary budgetary reallocations occur, the relative configuration of revenue shares, expenditure priorities, and staffing composition tends to remain structurally coherent.

Furthermore, longitudinal analyses of hospital financial statements in several European contexts indicate that the pandemic period did not generate statistically significant reclassification of hospitals into new financial typologies [125,126,127]. While revenue volatility increased in 2020–2021, clustering analyses conducted in Germany and Spain show persistent typological patterns when comparing pre- and post-pandemic years [128,129,130]. This suggests that financing regimes reflect deeper institutional logics rather than conjunctural fiscal fluctuations.

Within this conceptual and empirical framework, the expectation of temporal stability becomes analytically grounded. If financing regimes are structurally rooted in institutional rules, contractual design, and organizational routines, their configuration should remain broadly stable across pre-pandemic, pandemic, and post-pandemic periods [131,132,133]. Crisis timing may introduce temporary financial adjustments, but it is unlikely to generate systematic redefinition of regime membership. Accordingly, the following hypothesis is advanced:

**H2.**  *Identified financing regimes remain structurally stable across pandemic and post-pandemic periods, rather than being redefined by crisis timing.*

This hypothesis emerges at the intersection of institutional persistence, empirical observations from European financing reforms during the COVID-19 period, and contemporary resilience theory. Highly regulated public sectors such as healthcare tend to display significant structural continuity, as financing architectures are embedded in legal frameworks, contractual routines, and administrative norms that are not easily displaced by short-term shocks [134,135]. Evidence from European hospital systems during the pandemic suggests that emergency funding mechanisms were predominantly superimposed onto existing reimbursement structures, rather than replacing their core logic [136,137,138]. At the same time, resilience scholarship increasingly interprets crisis response as a process of absorption and stabilization within established institutional arrangements, rather than as structural redesign. In this light, testing H2 provides an opportunity to assess whether the pandemic constituted a genuine institutional break or whether its effects were filtered through and ultimately contained by pre-existing financing configurations.

### 2.3. Financing Composition and Case-Mix Complexity

Hospital case-mix complexity, commonly operationalized through the Case-Mix Index (*CMI*) within DRG-based reimbursement systems, captures the relative clinical intensity and resource requirements of treated patients [54,139]. Although *CMI* is frequently interpreted as a proxy for patient severity alone, a substantial body of research in health economics demonstrates that it is also shaped by organizational behavior and financial incentives [21,140]. In other words, case-mix complexity is not purely an exogenous clinical outcome; it is partially structured by the institutional environment in which hospitals operate. Financing composition, defined by the relative weight of contract-based revenues, public subsidies, and expenditure allocation priorities, plays a critical role in shaping this environment.

The theoretical linkage between financing composition and case-mix complexity can be traced to principal-agent models of provider behavior under prospective payment systems [54,141]. Under DRG-based or contract-based reimbursement, hospitals receive payments tied to coded diagnostic categories, which embed explicit financial incentives. Empirical research from Germany, France, and the Nordic countries has shown that hospitals more deeply integrated into contract-based reimbursement schemes tend to exhibit higher average *CMI* levels, reflecting either genuine specialization in more complex cases or more intensive coding practices [142,143,144]. Specialized studies [109,145,146], for example, document how the expansion of DRG-based payment systems has changed hospital activity patterns and stimulated alignment between case portfolio and reimbursement structure.

Conversely, hospitals that rely more heavily on fixed subsidies or historical budget allocations often face weaker marginal incentives to adjust case composition in response to complexity-weighted payments. The literature examining budget-based systems in Southern and Eastern Europe indicates that where funding is less tightly linked to case-based output, hospitals may display lower sensitivity of case-mix to reimbursement incentives [46,147,148]. This does not imply inefficiency; rather, it suggests that financing structure shapes the strategic orientation of clinical activity.

Expenditure composition further reinforces these dynamics. A high share of personnel expenditures may signal labor-intensive service delivery, but it does not automatically translate into higher DRG-weighted complexity [149,150]. Studies in the United Kingdom and Italy have shown that personnel intensity can coexist with relatively stable or moderate case-mix levels when service specialization remains limited [151,152,153]. By contrast, higher capital investment shares, particularly in infrastructure supporting specialized treatment units, have been associated with the capacity to manage more complex clinical cases. Research on hospital specialization patterns demonstrates that institutions with stronger investment capacity often concentrate higher-acuity patients, which is reflected in elevated *CMI* values [14,154,155].

In psychiatric services, the relationship between financing composition and complexity may be especially pronounced. Psychiatric case-mix is influenced not only by diagnostic severity but also by length-of-stay policies, staffing ratios, and the availability of specialized subunits (e.g., acute care, pediatric psychiatry, neurotic disorders) [156,157]. Because psychiatric care is labor-intensive and often characterized by extended treatment episodes, revenue stability and contract design can significantly affect institutional capacity to manage complex cases. Studies examining mental health reimbursement reforms in Western Europe suggest that integration into performance-based contracts correlates with increased differentiation in service mix and intensity [147,158].

Moreover, organizational theory emphasizes that financial structures shape long-term capacity-building decisions. Hospitals embedded in stronger contractual frameworks may invest in coding expertise, specialized clinical teams, and service differentiation strategies aligned with reimbursement incentives [146,159,160]. Over time, these investments generate cumulative effects that reinforce higher case-mix intensity. In contrast, institutions constrained by rigid expenditure ceilings or heavily subsidy-dependent financing may prioritize stability over specialization, resulting in different case portfolio structures.

Recent empirical contributions reinforce this structural perspective. Analyses of hospital panel data in Germany and Spain show statistically significant associations between revenue composition variables and the *CMI*, even after controlling for demographic and regional factors [161,162,163]. Similarly, studies in Central and Eastern Europe indicate that variation in contract integration explains a non-trivial share of cross-sectional differences in case-mix complexity [139,164,165]. These findings suggest that financing composition exerts an independent influence beyond short-term demand fluctuations or exogenous shocks.

Comparable relationships have also been documented across a wide range of non-European health systems, suggesting that the link between financing structures and hospital complexity is not context-specific but institutionally embedded. Specialized studies in the United States indicates that DRG-based and prospective payment systems shape not only coding practices but also the strategic configuration of case portfolios, as hospitals respond to complexity-weighted incentives embedded in reimbursement schemes [166,167]. In this context, variations in observed case-mix often reflect a combination of clinical factors and financially driven organizational behavior.

Similarly, research from emerging and transitional health systems [168,169,170], including China and Brazil, shows that hybrid financing arrangements—combining public funding, social health insurance, and private payments—play a decisive role in structuring hospital specialization, resource allocation patterns, and case-mix intensity. Despite ongoing reforms and institutional volatility in these settings, financing composition continues to exert a persistent influence on hospital behavior, reinforcing stable patterns of service differentiation and clinical complexity.

Taken together, this cross-country evidence points to a broader institutional mechanism through which financing architectures shape hospital performance. Rather than being a feature specific to European health systems, the relationship between financing composition and clinical complexity appears to reflect a generalizable logic of incentive-driven organizational adaptation, in which reimbursement structures systematically influence both the organization of care and the measured intensity of clinical activity.

Importantly, this literature also cautions against interpreting *CMI* exclusively as a measure of patient severity. Coding intensity, institutional specialization, and reimbursement incentives interact to shape observed case-mix levels [171,172]. Financing composition thus becomes a structural determinant of how clinical complexity is recorded, managed, and strategically organized within hospitals. Within this conceptual and empirical context, the following hypothesis is formulated:

**H3.**  *Structural financing composition significantly explains variation in hospital case-mix complexity across psychiatric institutions.*

This hypothesis is grounded in a cumulative body of theoretical and empirical insights suggesting that hospital case-mix complexity is shaped not only by clinical demand but also by the incentive structures embedded in financing arrangements. Prospective payment systems inherently connect revenue generation to complexity-weighted activity, thereby encouraging alignment between case portfolios and reimbursement design. At the same time, the internal allocation of expenditures conditions the institutional capacity for specialization, staffing configuration, and case management intensity, influencing how complexity is operationalized in practice. Empirical evidence from several European health systems further indicates that variation in revenue composition and expenditure priorities is statistically associated with differences in *CMI* levels across hospitals [73,127,173]. Within this framework, testing H3 enables a structured evaluation of whether psychiatric hospital complexity is primarily a reflection of patient-level characteristics or whether it is systematically embedded within differentiated financing architectures.

### 2.4. Pandemic Timing Versus Structural Determinants

The COVID-19 pandemic has generated a substantial body of research examining its impact on hospital activity, service utilization, and financial stability [174,175,176,177]. Many studies [178,179,180] documented abrupt declines in elective admissions, temporary shifts in case composition, and emergency reallocations of resources. In several European countries, psychiatric services experienced disruptions in outpatient care, modifications in referral pathways, and fluctuations in inpatient admissions during 2020–2021 [181,182,183]. However, while these descriptive findings highlight short-term turbulence, they do not necessarily establish that pandemic timing exerted an independent structural effect on case-mix complexity once institutional financing configurations are taken into account.

A central analytical distinction must therefore be drawn between unconditional temporal effects and conditional structural effects. Unconditional analyses compare pre-pandemic and pandemic averages without controlling for institutional heterogeneity. Conditional analyses, by contrast, assess whether pandemic timing retains explanatory power after structural determinants, such as revenue composition, expenditure allocation, and workforce configuration, are incorporated into the model. The latter approach aligns more closely with institutional economics and organizational theory, which posit that structural incentives mediate the impact of external shocks.

Empirical research across European hospital systems suggests that many observed pandemic effects attenuate once structural controls are introduced [184,185,186]. Studies have shown that although the total number of hospital admissions declined during the peak waves of COVID-19, the distribution of cases weighted by complexity remained significantly influenced by hospital specialization and funding integration. In DRG-based systems, complexity indices often rebounded to pre-pandemic trajectories once elective restrictions were lifted. Similar patterns were observed in various states, where hospital-level analyses showed that structural characteristics, particularly contractual integration and regional capacity, explained a greater share of case variation than dummy variables for the pandemic period [142,187,188].

In the mental health domain, several studies report temporary shifts in service modality, such as the expansion of telepsychiatry or reduced inpatient occupancy [116,189]. However, longitudinal analyses of psychiatric inpatient data in Northern Europe indicate that case severity distributions remained relatively stable across pre- and post-pandemic periods once institutional fixed effects were considered [190,191,192]. This finding suggests that while service delivery modalities adapted, the underlying structural determinants of complexity persisted.

Resilience theory further supports this interpretation. Contemporary scholarship distinguishes between “transformative resilience,” involving structural redesign, and “absorptive resilience,” characterized by stabilization within existing institutional frameworks [193,194]. Most European health systems appear to have exhibited the latter form during the pandemic: emergency funding measures were layered onto established reimbursement systems, and temporary compensations aimed to preserve institutional continuity rather than alter financing architecture. Consequently, pandemic timing may capture transient operational adjustments rather than durable structural change.

Moreover, statistical modeling studies emphasize the importance of avoiding over-attribution of temporal shocks when structural heterogeneity is present [195,196]. When hospital-level fixed effects or financing variables are omitted, period dummies can absorb variation that is in fact attributable to persistent institutional differences [197,198]. Once financing composition and expenditure structure are introduced, the independent effect of pandemic timing often declines or becomes statistically insignificant. This pattern has been documented in analyses of hospital productivity, coding intensity, and resource utilization across multiple OECD countries. Within this analytical context, the following hypothesis is formulated:

**H4.**  *Pandemic and post-pandemic timing have limited independent effects on case-mix complexity once structural financing determinants are controlled for.*

The hypothesis does not deny the existence of pandemic-related disturbances. Rather, it posits that these disturbances are mediated by, and embedded within, pre-existing financing regimes. If structural determinants constitute the primary organizing logic of hospital activity, then temporal indicators (PAN and POST) should exhibit limited explanatory power once revenue shares and expenditure allocations are accounted for. Testing H4 therefore enables a rigorous distinction between shock-driven variation and structurally embedded institutional behavior.

Taken together, the literature suggests that hospital case-mix complexity is shaped by a combination of structural financing factors, internal resource allocation patterns, workforce composition, and operational pressure indicators, which should therefore be explicitly reflected in the empirical specification.

*Research Gap*

Despite the rapid expansion of post-pandemic research on hospital performance, important analytical gaps remain insufficiently addressed. A substantial share of the literature examining COVID-19 effects relies on descriptive before–after comparisons or on empirical specifications that do not incorporate detailed structural controls. In such settings, observed temporal shifts may conflate genuine shock-induced variation with persistent institutional heterogeneity, thereby obscuring the relative weight of structural determinants. At the same time, research focusing on financing composition and case-mix complexity has frequently developed independently of pandemic-oriented analyses, treating institutional architecture and crisis timing as separate domains of inquiry rather than as interacting dimensions within a unified framework.

An additional limitation concerns the geographical concentration of existing evidence. Quantitative panel analyses linking hospital financing structures to complexity indicators are disproportionately derived from Western European or high-income OECD systems, where data infrastructures are well established and payment reforms have been extensively studied. By contrast, Central and Eastern European health systems, particularly in the field of psychiatric services, remain comparatively underrepresented in longitudinal, hospital-level empirical research. This imbalance constrains the external validity of broader theoretical claims regarding financing architecture and institutional resilience.

Beyond these contextual gaps, the prevailing methodological orientation of the literature has tended to remain variable-centric. Much of the empirical work isolates individual coefficients, such as the marginal effect of a revenue share or a pandemic dummy, without identifying whether hospitals operate within coherent, internally consistent financing configurations. The absence of regime-based typologies limits the ability to interpret institutional heterogeneity as structured and systematic rather than as residual statistical noise. Only a limited number of studies integrate multivariate classification techniques with outcome modeling in order to connect structural typologies to measurable clinical indicators. These gaps also carry important implications for policy and practice. Without a clearer understanding of how financing regimes shape hospital behavior and case-mix complexity, policymakers may rely on uniform financing reforms that fail to account for institutional heterogeneity and may unintentionally reinforce regional or organizational disparities. In the field of mental health services, where clinical needs are complex and resource allocation is highly sensitive to institutional design, the absence of actionable evidence on financing structures limits the capacity of both decision-makers and hospital managers to develop targeted, efficient, and equitable interventions.

The present study responds to these shortcomings by adopting an integrated analytical design that simultaneously identifies structurally differentiated financing regimes, evaluates their temporal stability across pre-pandemic, pandemic, and post-pandemic periods, and estimates their explanatory relevance for case-mix complexity within a multivariate framework that explicitly controls for temporal indicators. By situating this approach within psychiatric hospitals in a Central and Eastern European setting, the analysis contributes novel empirical evidence to international debates on health system resilience, institutional persistence, and the structural determinants of hospital complexity.

*Concluding Remarks on the Literature*

The literature reviewed above converges on a central insight: hospital performance, including case-mix complexity, is shaped by institutional financing architecture as much as by clinical demand or exogenous shocks. Mixed financing arrangements generate heterogeneous configurations that may crystallize into persistent regimes. Institutional theory and resilience scholarship suggest that these regimes exhibit structural continuity even under crisis conditions. Empirical studies across Europe further indicate that financing composition and expenditure priorities are systematically associated with variation in the *CMI*, while pandemic timing effects tend to diminish once structural controls are introduced.

However, these strands of research have rarely been integrated within a single analytical design capable of distinguishing regime-based heterogeneity from temporal shock effects in psychiatric services. By articulating and empirically testing H1–H4, the present study positions itself at this intersection, advancing a structurally grounded interpretation of hospital case-mix dynamics. The following sections translate these theoretical expectations into an empirical strategy capable of evaluating whether psychiatric hospital complexity is primarily shock-driven or structurally embedded within differentiated financing regimes. From a policy and managerial perspective, this literature also indicates that financing architecture should not be treated as a neutral administrative background, but as an active determinant of hospital behavior, service differentiation, and resource allocation. A more nuanced understanding of financing regimes may therefore support the design of reimbursement systems better aligned with clinical needs, help hospital managers optimize staffing and expenditure priorities, and provide policymakers with a stronger basis for developing differentiated mental health policies adapted to institutional and regional diversity.

## 3. Materials and Methods

### 3.1. Data Collection, Ethical Considerations, and Dataset Structure

The empirical analysis is based on administrative panel data collected for 126 public and private healthcare institutions in Romania over the period 2019–2024. The unit of observation is the hospital section–year, with the dataset covering psychiatric services across all eight Romanian development regions within a common national regulatory and financing framework [199]. The database includes annual aggregated information on clinical activity, financial structure, expenditure composition, workforce characteristics, and operational workload. The dataset contains indicators related to confirmed cases, the Case-Mix Index (*CMI*), total cost per patient (TCP), revenue sources, expenditure categories, staffing structure, and service capacity, allowing for the construction of the structural and outcome variables used in the empirical analysis.

All data are reported at institutional and section level and originate from official administrative reporting sources [200]. No individual-level patient information, personal identifiers, or other sensitive data were included. The study relies exclusively on anonymized aggregated administrative records and does not involve human subjects research as defined under EU Regulation 2016/679 (GDPR), nor does it require individual informed consent. The analytical dataset can be made available upon reasonable request to the corresponding author, subject to institutional approval and applicable data sharing regulations.

### 3.2. Variable Construction and Operationalization

To ensure conceptual clarity and analytical consistency, all variables included in the empirical analysis are summarized in Table 1. The study distinguishes between outcome variables, structural financing indicators, expenditure composition measures, workforce structure variables, operational pressure indicators, and temporal controls. The primary dependent variable is the Case-Mix Index (*CMI*), which captures DRG-based clinical complexity, complemented by Total Cost per Patient (TCP) and the number of confirmed cases (CASEs) as additional outcome and scale indicators. The selection of explanatory variables is explicitly grounded in the existing literature on health economics, hospital financing, and institutional theory, ensuring that all relevant structural drivers of hospital performance and case-mix complexity are systematically captured. In line with prior empirical and theoretical contributions, the explanatory variables are organized into four core dimensions reflecting the institutional configuration of hospitals: financing structure, expenditure allocation, workforce composition, and operational pressure.

The explanatory variables reflect the structural configuration of hospital financing and organization and are selected based on established findings in the health economics and hospital performance literature, ensuring that the empirical specification captures the main institutional and operational drivers of case-mix complexity.

The financing structure is operationalized through the share of revenues from health insurance contracts (SH_CNAS), the share of public subsidies (SH_GRANTS), and the share of current revenues (SH_VCUR). The share of revenues from health insurance contracts (SH_CNAS) captures the degree of integration into contract-based reimbursement systems and is widely associated with incentive intensity, DRG-related behavior, and case-mix configuration [55,201]. The share of public subsidies (SH_GRANTS) reflects reliance on non-contractual public transfers, typically linked to softer budget constraints and reduced marginal incentives [50,202]. The share of current revenues (SH_VCUR) captures the overall weight of recurrent funding streams, reflecting financial stability and operational continuity [31,203].

The expenditure structure is captured through the share of personnel expenditures (SH_PERS), the share of goods and services expenditures (SH_GDS), the share of capital expenditures (SH_CAPEX), and the share of current expenditures (SH_CHELT_CUR). The share of personnel expenditures (SH_PERS) reflects labor intensity, which is particularly relevant in psychiatric services characterized by high dependence on human resources [204,205]. The share of goods and services expenditures (SH_GDS) captures operational expenditure intensity related to service delivery processes [124,206], The share of capital expenditures (SH_CAPEX) reflects investment capacity and infrastructure development, which are associated in the literature with hospital specialization and the ability to manage more complex clinical cases [150,207]. The share of current expenditures (SH_CHELT_CUR) captures the predominance of current expenditure commitments, reflecting the balance between recurrent operational spending and long-term investment capacity [73,208].

The workforce structure is operationalized through the share of psychiatrists (SH_PSYCH), the share of medium-level healthcare staff (SH_MED), and the share of auxiliary personnel (SH_AUX). The share of psychiatrists (SH_PSYCH) captures the availability of specialized medical expertise, which is directly linked to clinical complexity and treatment capacity [209,210]. The share of medium-level healthcare staff (SH_MED) reflects the contribution of nursing and intermediate staff, which play a key role in care delivery [211,212]. The share of auxiliary personnel (SH_AUX) captures support staff intensity, influencing operational efficiency and organizational structure [213,214].

Operational pressure is proxied by the number of cases per bed (CASES_PER_BED) and the number of cases per physician (CASES_PER_MED), which measure workload intensity and capacity constraints. The number of cases per bed (CASES_PER_BED) reflects infrastructure utilization pressure [124,215], while the number of cases per physician (CASES_PER_MED) captures staff workload intensity [216,217], both being widely used indicators in hospital efficiency and performance studies.

Temporal dynamics are incorporated through two dummy variables: pandemic period (PAN), capturing the period 2020–2021, and post-pandemic period (POST), reflecting the period 2022–2024, with 2019 serving as the baseline year. These variables allow for the identification of exogenous shock effects and the distinction between structural determinants and crisis-induced variation.

The dependent variables included in the empirical analysis are the Case-Mix Index (*CMI*), reflecting DRG-based clinical complexity, the total cost per patient (TCP), capturing resource intensity, and the number of confirmed cases (CASEs), reflecting activity volume. These variables allow for the joint assessment of clinical complexity, cost dynamics, and service scale within the hospital system.

To ensure comparability across heterogeneous indicators and to avoid scale dominance in multivariate procedures, all variables included in the clustering analysis were standardized using z-score transformation prior to estimation (Appendix A.1).

### 3.3. Empirical Strategy and Statistical Procedures

Given the objectives of this study, which aim to identify latent structural configurations of hospital financing and organization, a multivariate clustering approach is particularly appropriate. Unlike variable-centered methods, cluster analysis allows for the identification of internally coherent groups of hospital units characterized by similar patterns of revenue composition, expenditure allocation, workforce structure, and operational pressure. This enables the operationalization of “financing regimes” as empirical constructs and directly supports the identification of structural typologies (RQ1), which subsequently form the basis for testing temporal stability (RQ2) and outcome differences in case-mix complexity (RQ3–RQ4).

The empirical strategy follows a sequential design combining multivariate classification, temporal validation, and outcome modeling in order to distinguish structural financing regimes from pandemic-related effects.

The structural typologies of psychiatric hospitals are identified using a two-step clustering procedure. All variables included in the clustering analysis (financing structure, expenditure composition, workforce structure, and operational pressure indicators) were standardized using z-score transformation:

(1) $$Z(X_{it})=\frac{X_{it}-\bar{X}}{s_{X}}$$

where $X_{it}$ denotes the value of structural indicator X for hospital section i in year t, $\bar{X}$ is the sample mean, and $s_{X}$ is the standard deviation.

A hierarchical clustering algorithm based on Ward’s method and squared Euclidean distance was initially applied to determine the optimal number of clusters. The inspection of the dendrogram and agglomeration schedule indicated a three-cluster solution, which was subsequently refined using K-means clustering. The resulting categorical variable, CLUSTER_FIN, captures distinct financing–organizational regimes.

Also, the temporal robustness of the identified clusters is assessed. The association between cluster membership and pandemic timing (PAN and POST) is examined using cross-tabulations and Pearson’s chi-square test:

(2) $$\chi^{2}=\sum \frac{\left(O_{jk}-E_{jk}{)}^{2}\right.}{E_{jk}}$$

where $O_{jk}$ represents the observed frequency in cell jk, and $E_{jk}$ denotes the expected frequency under independence. Given the descriptive nature of this test, a more stringent validation is implemented through a split-sample approach, whereby clustering is estimated separately for the pre-pandemic and post-pandemic subsamples. The similarity between the resulting partitions is evaluated using the Adjusted Rand Index (*ARI*), which corrects for chance agreement. In addition, a centroid displacement analysis is conducted to assess the stability of cluster positions in the multidimensional space, based on Euclidean distances between matched centroids across periods.

The identified regimes are linked to clinical and financial outcomes. Differences in case-mix complexity are examined using a factorial ANOVA specification:

(3) $$CMI_{it}=\mu +\alpha_{c(i)}+\beta_{p(t)}+(\alpha \beta {)}_{c(i)p(t)}+\varepsilon_{it}$$

where $CMI_{it}$ represents the Case-Mix Index of hospital unit i in year t; μ is the overall mean; $\alpha_{c(i)}$ denotes the fixed effect associated with cluster membership c; $\beta_{p(t)}$ represents the effect of pandemic status p(PAN); and $(\alpha \beta {)}_{c(i)p(t)}$ captures the interaction between structural regime and pandemic period. The error term $\varepsilon_{it}$ reflects unexplained variance.

To further assess the determinants of case-mix complexity, a multivariate regression model is estimated:

(4) $$CMI_{it}=\beta_{0}+\beta_{1}PAN_{t}+\beta_{2}POST_{t}+\beta_{3}SH\_CNAS_{it}+\beta_{4}SH\_GRANTS_{it}+\beta_{5}SH\_VCUR_{it}+\beta_{6}SH\_PERS_{it}+\beta_{7}SH\_GDS_{it}+\beta_{8}SH\_CAPEX_{it}+\varepsilon_{it}$$

where

– $CMI_{it}$ represents the Case-Mix Index of hospital unit i in year t, capturing DRG-based case complexity;
– $\beta_{0}$ is the intercept, reflecting baseline case-mix in the reference year (2019);
– $PAN_{t}$ is a dummy variable equal to 1 during the pandemic period (2020–2021) and 0 otherwise;
– $POST_{t}$ is a dummy variable equal to 1 during the post-pandemic period (2022–2024) and 0 otherwise;
– $SH\_CNAS_{it}$ measures the share of revenues obtained through national health insurance contracts in total revenues;
– $SH\_GRANTS_{it}$ captures the proportion of subsidies and public transfers in total revenues;
– $SH\_VCUR_{it}$ reflects the share of current revenues in total revenues;
– $SH\_PERS_{it}$, $SH\_GDS_{it}$, and $SH\_CAPEX_{it}$ represent the allocation of total expenditures toward personnel, goods and services, and capital investment, respectively;
– $\varepsilon_{it}$ denotes the error term.

This specification incorporates both temporal indicators and structural financing variables, allowing for the identification of conditional effects and disentangling structural determinants from crisis-related variation. All estimations were conducted using IBM SPSS Statistics (version 26) and STATA (version 19). Statistical inference is based on conventional significance thresholds (*p* < 0.05), with effect sizes and model explanatory power evaluated jointly.

The empirical strategy outlined above enables a systematic identification of structural financing regimes, their temporal validation, and their linkage to clinical complexity outcomes. The following section presents the empirical results in a sequential manner, directly reflecting this analytical design and allowing for a coherent interpretation of regime-based differences and temporal dynamics.

## 4. Results

This section reports the empirical results in accordance with the sequential research design, moving from structural regime identification and temporal validation to outcome modeling, with explicit reference to the formulated hypotheses.

### 4.1. Sample Structure and Dispersion in Financing–Organization Indicators

The analytical sample comprises 752 hospital section–year observations for 2019–2024, covering psychiatric units across Romania’s eight development regions. The indicators used to identify financing–organization regimes display substantial cross-sectional variation in revenue composition, expenditure allocation, workforce structure, and operational pressure, which empirically supports both variable standardization and the use of multivariate clustering techniques.

At the aggregate level, the descriptive statistics indicate meaningful heterogeneity in the relative weight of CNAS revenues, grants and subsidies, current revenues, personnel expenditures, goods and services, capital expenditures, staff composition, and workload indicators. This variation suggests that psychiatric hospital units do not operate under a uniform institutional profile, but rather under differentiated structural configurations that may be expected to generate distinct financing–organization regimes.

Regional descriptive statistics, reported in Appendix A.2, further confirm that this heterogeneity is not random, but territorially patterned. Some regions combine relatively higher case-mix values with stronger workload pressure, while others display lower complexity and more balanced operational ratios. These differences provide additional support for the view that structural diversity within the Romanian psychiatric hospital system is persistent and analytically relevant.

### 4.2. Identification of Structural Financing Regimes: Hierarchical Clustering and K-Means Refinement

To uncover persistent financing–organization typologies, the first step applied hierarchical clustering using Ward’s linkage and squared Euclidean distance on standardized indicators capturing revenue composition, expenditure structure, workforce mix, and operational pressure. The hierarchical solution supported a three-cluster structure, which was then refined through K-means to obtain a stable partition of observations into internally coherent regimes.

**Figure 1:**
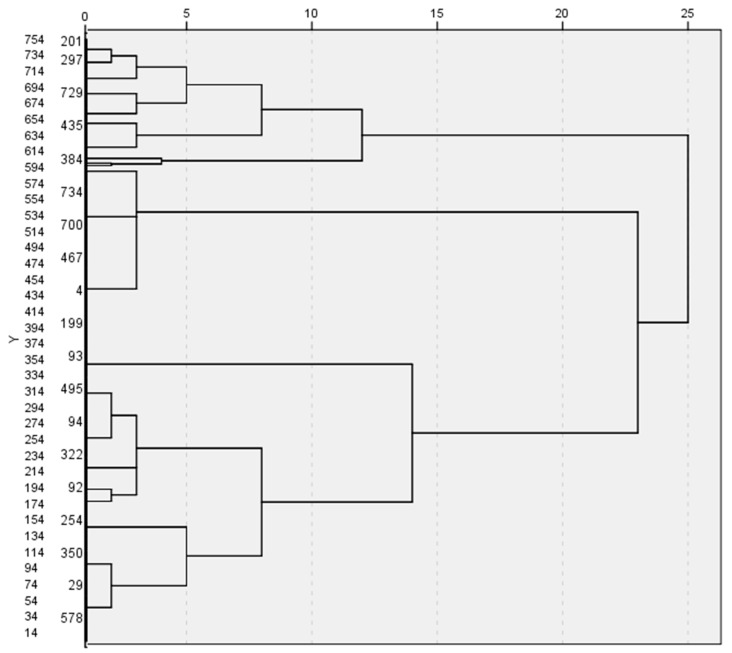
Dendrogram of hierarchical clustering using Ward’s method.

To enhance the transparency and robustness of the clustering procedure, the hierarchical clustering results are illustrated through the dendrogram presented in Figure 1, which visually supports the three-cluster solution based on Ward’s linkage and squared Euclidean distance.

The dendrogram reveals a clear separation of observations into three major clusters, with a substantial increase in linkage distance beyond this partition, confirming the appropriateness of the three-regime solution retained for subsequent analysis.

The resulting distribution is uneven but highly interpretable in institutional terms: Cluster 2 represents the dominant regime (430 observations; 57.2%), Cluster 3 forms a substantial secondary regime (292 observations; 38.8%), while Cluster 1 is a small but clearly differentiated group (30 observations; 4.0%) consistent with an “extreme configuration” rather than random noise (Table 2).

Rather than conceptualizing the pandemic as a uniform exogenous shock generating homogeneous institutional responses, the empirical evidence reveals that Romanian psychiatric hospitals operate within differentiated, path-dependent financing and organizational regimes that precede the crisis and continue to shape institutional dynamics beyond it. This structural persistence lends substantive support to the paper’s typology-driven argument (H1), highlighting the primacy of pre-existing regime configurations over purely temporal shock effects (Table 3).

The standardized cluster centroids reported in Table 3 confirm the existence of three structurally distinct financing–organization regimes, differentiated primarily by workload intensity, personnel composition, and expenditure priorities, thereby substantiating the typology-based interpretation advanced in this study.

To further assess the stability and consistency of the clustering solution, a cross-classification analysis was conducted between the hierarchical clustering results and the K-means partition (k = 3).

The results are reported in Table 4.

The cross-classification results indicate a substantial degree of agreement between the hierarchical and K-means clustering solutions, as reflected by the concentration of observations along the main diagonal. In particular, Cluster 2 and Cluster 3 exhibit strong consistency across methods, while limited reallocation is observed for a subset of observations in Cluster 2, suggesting the presence of borderline units. Overall, the results confirm the robustness and stability of the identified three-cluster structure.

This validation step reduces the risk of method-dependent classification bias and strengthens the interpretability of the identified financing regimes.

### 4.3. Temporal Robustness: Cluster Regimes Are Not “Pandemic-Made”

A central validation step examined whether cluster membership is mechanically associated with the pandemic period. Cross-tabulations of cluster assignment with PAN and POST indicate that all three regimes are represented both before and during/after the pandemic, with comparable within-cluster proportions across periods. The chi-square test of independence (Table 5) confirms the absence of a statistically significant association between cluster membership and PAN (χ^2^(2) = 1.819, *p* = 0.403), suggesting that the identified regimes are not directly driven by crisis timing.

However, as such cross-tabulations remain descriptive, a more stringent validation was implemented through a temporal split-sample approach. The clustering procedure was estimated separately for the pre-pandemic and post-pandemic subsamples using identical specifications, and the resulting partitions were compared using the Adjusted Rand Index (*ARI*). The computed *ARI* value of 0.269 indicates a low-to-moderate level of agreement between the two clustering solutions, pointing to partial structural continuity combined with non-negligible reallocation of units across regimes. Table 6 reports the contingency matrix between the pre-pandemic and post-pandemic cluster assignments.

The correspondence matrix reveals a partial structural alignment rather than one-to-one mapping between regimes. While the first pre-pandemic cluster is largely absorbed into a single post-pandemic configuration, the second and third clusters exhibit substantial redistribution across post-pandemic regimes. This fragmentation explains the moderate *ARI* value and indicates that the pandemic period acted not only as a stress test but also as a reconfiguration mechanism of existing institutional patterns.

The *ARI* test was computed using the standard formulation as follows:

(5) ARI=∑ijnij2−∑iai2∑jbj2n212∑iai2∑jbj2−∑iai2∑jbj2n2

where $n_{ij}$ denotes the contingency counts, $a_{i}$ and $b_{j}$ are cluster marginals, and n is the total number of observations (*n* = 752). The adjustment for chance ensures that the index equals zero under random partitioning and one under perfect agreement.

To complement the split-sample validation based on the Adjusted Rand Index, a centroid displacement analysis was conducted in order to assess whether the structural position of the clusters in the multidimensional space remains stable across periods.

The test involves computing the centroids (mean standardized values of the clustering variables) for each cluster in the pre-pandemic sample and comparing them with the corresponding centroids in the post-pandemic sample. The degree of structural change is quantified using the Euclidean distance between matched centroids:

(6) $$D_{k}=\sqrt{\sum_{m=1}^{M}{\left({\bar{x}}_{km}^{pre}-{\bar{x}}_{km}^{post}\right)}^{2}}$$

where $D_{k}$ denotes the displacement of cluster k, and ${\bar{x}}_{km}$ represents the centroid value for variable m in cluster k.

Based on the maximum overlap derived from the contingency matrix, clusters were matched across periods, and centroid distances were computed. The results indicate a heterogeneous pattern of structural change, with displacement values ranging from approximately 1.19 to 4.20.

Specifically, the cluster corresponding to PRE 2 → POST 1 exhibits the lowest displacement (*D* ≈ 1.19), indicating relatively strong structural continuity. In contrast, the cluster corresponding to PRE 3 → POST 3 shows moderate displacement (*D* ≈ 1.70), suggesting partial reconfiguration. The largest shift is observed for the cluster corresponding to PRE 1 → POST 3 (*D* ≈ 4.20), indicating a more substantial change in its multidimensional profile. These results point to asymmetric structural dynamics across clusters, where regimes remain broadly comparable in relative terms. This pattern is consistent with the *ARI* results, suggesting that temporal changes are driven not only by reallocation of units across clusters, but also by varying degrees of transformation in cluster profiles. The detailed cross-tabulations is reported in Appendix A.3.

Taken together, these results refine the interpretation of H2. While the absence of a significant association between cluster membership and pandemic timing supports the idea that regimes are not purely crisis-induced, both the *ARI* and centroid displacement analyses indicate that temporal stability is only partial. The identified regimes persist as recognizable structural configurations, but their internal composition and relative positioning evolve over time. Accordingly, H2 is only partially supported, with the evidence pointing to adaptive reconfiguration rather than strict invariance.

### 4.4. Regime-Linked Differences in Case-Mix Complexity and Costs

Having identified persistent regimes, the next step links typologies to clinical complexity (*CMI*) and resource intensity (TCP).

From a theoretical perspective, case-mix complexity is shaped by both structural and contextual factors, including financing arrangements, resource allocation mechanisms, and institutional organization, as well as exogenous shocks such as health crises. In this framework, the cluster-based typology captures latent structural configurations that integrate multiple dimensions of hospital functioning, allowing for a reduced-form representation of otherwise unobservable institutional drivers.

Mean comparisons indicate non-trivial differences in average *CMI* across regimes: Cluster 1 has the highest mean *CMI* (1.607; SD = 0.160; N = 30), Cluster 2 follows (1.570; SD = 0.248; N = 430), and Cluster 3 shows the lowest mean *CMI* (1.483; SD = 0.276; N = 292). The ordering is consistent with the interpretation that structurally “high-pressure” units and/or configurations with tighter staffing constraints may concentrate more complex cases or reflect higher DRG-weighted intensity in recorded activity. A complementary perspective emerges from TCP: Cluster 2 records the highest mean TCP (1661.684; SD = 184.414), while Cluster 1 shows the lowest (1528.367; SD = 114.410), with Cluster 3 in between (1590.795; SD = 177.186). This pattern is analytically valuable because it cautions against equating “higher costs” with “higher case-mix” in a mechanical way; rather, it suggests that cost intensity may reflect expenditure structure and procurement/service delivery modalities (e.g., goods and services reliance) in addition to clinical complexity. The descriptive evidence is summarized in Table 7.

The post-pandemic period is associated with a higher average *CMI* at the aggregate level (POST = 1 mean 1.570 vs. POST = 0 mean 1.490), and simultaneously a higher average TCP (1694.479 vs. 1532.639). While informative, these descriptive contrasts must be situated within the structural heterogeneity of the system, since observed period differentials may be shaped not only by temporal dynamics but also by pre-existing financing arrangements and organizational configurations.

### 4.5. ANOVA Validation: Regime Effects Dominate Simple Pandemic Shifts

To formally assess whether *CMI* differs across regimes and whether the pandemic period modifies these differences, a two-way UNIANOVA was estimated with *CMI* as the dependent variable and CLUSTER_FIN, PAN, and their interaction as fixed factors. Results show a statistically significant main effect of financing regime on *CMI* (F = 5.845, *p* = 0.003), while PAN is not significant (F = 0.359, *p* = 0.549), and the interaction CLUSTER_FIN × PAN is also not significant (F = 0.822, *p* = 0.440). The explained variance of this specification is relatively modest (R^2^ = 0.043; adjusted R^2^ = 0.036), which is expected given the parsimonious design of the model and the fact that case-mix complexity is influenced by a wide range of clinical and demographic factors not directly observable in aggregate data (Table 8).

To ensure the robustness of the estimated model, a set of post-estimation diagnostics was conducted. The homogeneity of variance assumption was assessed using Levene’s test (Table 9), which was not statistically significant (F(5, 746) = 1.097, *p* = 0.361). This indicates that the variance in the *CMI* is comparable across groups, supporting the robustness of the ANOVA estimates.

**Figure 2:**
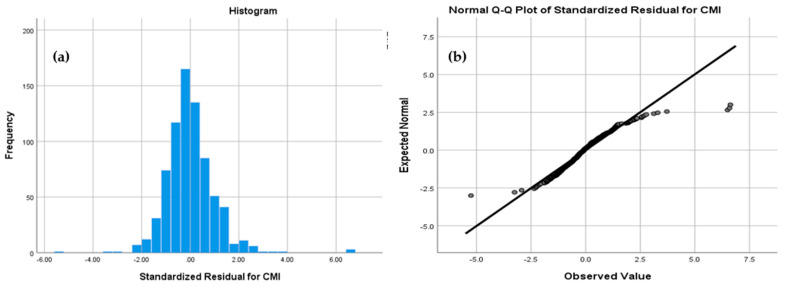
Distribution of standardized residuals ((**a**) histogram of standardized residuals with normal curve overlay; (**b**) normal Q–Q plot of standardized residuals).

The distribution of standardized residuals was examined using histogram and normal Q–Q plots (Figure 2), which indicate an approximately normal pattern, despite some deviations in the tails.

Formal normality tests (Kolmogorov–Smirnov and Shapiro–Wilk) were statistically significant (*p* < 0.001), which is expected given the large sample size (N = 752) and their sensitivity to minor deviations. Skewness values (1.203) and kurtosis values (8.237) suggest some degree of asymmetry and leptokurtosis; however, such deviations are not uncommon in applied health data and do not invalidate ANOVA results, particularly in large samples. Overall, the residual distribution can be considered sufficiently close to normal for reliable inference.

While the explanatory power of the model is modest (R^2^ = 0.043), this is consistent with the inherently multifactorial nature of case-mix complexity, which is influenced by numerous clinical and institutional determinants not fully captured in administrative datasets. The model is therefore intentionally parsimonious, aiming to isolate the structural effect of financing–organization regimes rather than to provide a fully specified predictive framework.

To ensure the appropriateness of the estimation strategy, additional panel data diagnostics were conducted (Table 10), taking into account the longitudinal structure of the dataset (126 hospitals observed over six years).

A Hausman specification test was first performed in order to compare fixed effects and random effects estimators. The results indicate that the null hypothesis of no systematic difference between estimators cannot be rejected (χ^2^ = 0.332, *p* = 0.988 for the *CMI* model; χ^2^ = 1.767, *p* = 0.880 for the TCP model), suggesting that the random effects specification is consistent and that unobserved individual effects are not correlated with the regressors. In addition, a Breusch–Pagan Lagrange Multiplier (LM) test for random effects was performed to assess whether panel estimation is preferred over pooled OLS. The results indicate that the null hypothesis of no panel effects is rejected (chibar^2^ = 15.79, *p* < 0.001), confirming the presence of significant unobserved heterogeneity across hospital units. Taken together, these results support the use of a random effects specification as the most appropriate panel estimator. However, given the primary objective of the study—namely to identify structural relationships associated with financing composition—and for comparability with the typology-based analysis, pooled OLS estimates are retained as a baseline specification, while panel-based estimators are used to confirm the robustness of the findings. These findings confirm that the empirical results are robust across alternative specifications and reflect stable structural associations rather than artifacts of model selection.

This result provides a robust empirical bridge from the typology analysis to the paper’s core argument and directly aligns with the logic of H4: “pandemic timing” is not the primary driver once structural configurations are taken into account; instead, persistent financing–organization regimes shape the baseline level of case-mix complexity, a finding that remains consistent across alternative specifications and diagnostic tests.

### 4.6. Structural Financing Configuration Outweighs Pandemic Timing in Explaining Case-Mix Complexity

The final multivariate specification estimates the determinants of case-mix complexity (*CMI*) at the national level, incorporating both temporal indicators (PAN, POST) and structural financing and expenditure variables. The model is statistically significant overall (F(8, 743) = 6.218, *p* < 0.001), with an R^2^ of 0.063 (adjusted R^2^ = 0.053), indicating that structural factors explain a non-negligible share of cross-sectional variation in the *CMI* (Table 11).

Consistent with the typology-based framework, pandemic timing does not emerge as a robust predictor of case-mix complexity. The PAN dummy is statistically insignificant, while POST shows only marginal significance, suggesting that temporal effects, once structural determinants are controlled for, do not systematically alter the DRG-based complexity of hospital activity.

By contrast, structural financing variables display more consistent explanatory power. The share of revenues derived from national health insurance contracts (SH_CNAS) has a positive and statistically significant effect, indicating that hospitals more deeply embedded in contract-based reimbursement regimes systematically exhibit higher case-mix complexity. In contrast, the share of personnel expenditures (SH_PERS) is negatively associated with *CMI*, suggesting that labor intensity alone does not translate into higher DRG-weighted complexity once structural and temporal factors are jointly considered. The remaining variables do not display statistically robust effects in the global specification, reinforcing the interpretation that variation in clinical complexity is driven by specific components of financing architecture rather than by broad expenditure aggregates.

To assess the robustness of these findings, region-specific regressions were estimated (Table 12).

The results reveal substantial territorial heterogeneity in coefficient magnitude and statistical significance. However, despite this variability, a consistent pattern emerges: structural financing variables retain explanatory relevance across most regions, while pandemic timing remains weak or insignificant.

This evidence indicates that regional differences reflect variations in the intensity and configuration of structural effects rather than a shift toward temporally driven dynamics. In some regions, case-mix complexity is strongly associated with revenue composition and capital intensity, while in others the relationship is weaker or more diffuse. Nevertheless, the dominance of structural determinants over pandemic timing remains a recurrent empirical pattern.

Overall, the results support H4 and reinforce the central argument of the study: psychiatric hospital case-mix complexity is primarily embedded in differentiated financing regimes, while pandemic effects, although observable at the descriptive level, do not constitute a dominant explanatory factor once structural characteristics are taken into account.

## 5. Discussion and Policy Implications

The findings of this study provide a structurally grounded reinterpretation of the relationship between financing architecture and clinical complexity in psychiatric hospitals operating within a centralized health insurance system. Contrary to narratives that attribute post-2020 shifts in hospital performance primarily to the COVID-19 shock, the empirical evidence demonstrates that case-mix complexity is predominantly shaped by persistent financing configurations rather than by temporary pandemic timing. This distinction is analytically important, because it reorients the explanatory focus from exogenous crisis events to endogenous institutional architecture. The identification of three structurally distinct financing–organization regimes confirms that psychiatric hospitals in Romania operate within differentiated institutional configurations, even under a formally unified national financing framework. The absence of a statistically significant association between cluster membership and pandemic timing further strengthens this interpretation: the regimes uncovered by the clustering procedure are not crisis-induced categories but rather entrenched structural typologies. In this respect, this study advances the literature on health system resilience by illustrating that resilience may manifest not only as adaptive reconfiguration but also as structural persistence under stress.

The multivariate results deepen this interpretation. The global regression model demonstrates that the share of contract-based revenues (SH_CNAS) is positively associated with case-mix complexity, while personnel expenditure shares exhibit a negative relationship. Pandemic timing variables do not retain robust explanatory power once structural controls are introduced. This finding is theoretically significant because it challenges the assumption that crisis periods necessarily produce lasting shifts in clinical workload patterns. Instead, the results suggest that the structural incentives embedded in financing composition play a more decisive role in shaping case-mix intensity.

From a comparative perspective, this contribution addresses a clear gap in the international literature. Much of the empirical evidence on hospital financing and DRG-based complexity originates from Western European systems or from high-income OECD contexts where detailed micro-level data are available. Central and Eastern European systems remain underrepresented in quantitative studies relying on administrative hospital-level panel data. By combining clustering techniques with multivariate regression on a multi-year national dataset, the present research extends empirical evidence to an institutional setting characterized by transitional financing arrangements and uneven territorial capacity. The study thus contributes to bridging the East–West empirical divide in health economics research.

Another important contribution lies in the integration of structural typology analysis with outcome modeling. Rather than examining financing variables in isolation, the study first identifies regime-based configurations and then links them to clinical outcomes. This two-step approach allows the analysis to move beyond variable-level associations toward a regime-based understanding of institutional behavior. In doing so, the paper aligns with institutionalist perspectives in public finance and organizational economics, which emphasize the role of structural arrangements over short-term shocks in explaining performance variation.

The regional robustness analysis further reinforces the structural interpretation. Although explanatory power varies across regions, financing composition consistently emerges as a key determinant of case-mix complexity. Even where coefficients differ in magnitude or direction, the dominance of structural variables over pandemic timing remains evident. This territorial heterogeneity underscores the importance of contextualized policy responses rather than uniform national interventions. The policy implications of these findings are substantial and extend beyond the Romanian case.

The strong association between contract-based revenue shares and case-mix complexity suggests that financing incentives embedded in health insurance contracts influence not only revenue stability but also the clinical composition of treated cases. Policymakers should therefore reassess the design of psychiatric service contracts to ensure that reimbursement formulas do not inadvertently encourage case selection behaviors or distort clinical prioritization. Rather than relying exclusively on volume-based or historical contract allocations, a more nuanced adjustment mechanism incorporating regional workload intensity and structural staffing ratios could enhance allocative fairness.

The negative association between personnel expenditure share and case-mix complexity raises questions about the efficiency and strategic alignment of staffing structures. This does not imply that higher staffing reduces complexity per se; rather, it suggests that expenditure concentration on personnel, without complementary investment in service diversification or infrastructure, may not translate into higher DRG-weighted activity. A potential policy direction would involve introducing performance-sensitive budgeting frameworks that link personnel expansion to demonstrable improvements in service mix complexity, rather than maintaining rigid expenditure ceilings detached from outcome indicators.

The regional heterogeneity observed in the robustness analysis indicates that a “one-size-fits-all” financing policy is unlikely to produce optimal outcomes. Regions exhibiting strong structural sensitivity to financing composition may benefit from targeted contractual recalibration, while regions displaying structural stability may require different levers, such as service integration mechanisms or coordinated referral networks. The findings therefore support a territorially differentiated policy approach within the broader national financing architecture.

The absence of a robust pandemic effect in the presence of structural controls suggests that crisis-related funding adjustments should not be interpreted as substitutes for long-term structural reform. Temporary infusions of resources during emergencies may stabilize operations, but they do not fundamentally alter the institutional drivers of clinical complexity. Sustainable reform must therefore address financing architecture rather than relying on episodic fiscal injections.

The identification of high-pressure regimes characterized by elevated cases per bed and per physician highlights the need for structural workload monitoring mechanisms. Instead of reactive staffing adjustments, policymakers could implement forward-looking capacity planning models integrating workload ratios, contract composition, and case-mix indicators to anticipate imbalances before they translate into quality deterioration or workforce burnout.

The policy implications emerging from these findings extend beyond conventional resource expansion strategies and instead point toward structural recalibration of financing architecture. Rather than focusing exclusively on aggregate funding increases or uniform staffing adjustments, the results indicate the need for incentive-consistent contract design, differentiated regional calibration of reimbursement parameters, and closer alignment between expenditure composition and case-mix intensity. In this framework, financing policy becomes a structural instrument shaping clinical configuration, not merely a fiscal envelope. Effective reform therefore requires integrating workload indicators, revenue composition, and expenditure structure into coordinated budgeting and contracting mechanisms capable of addressing territorially differentiated institutional configurations.

## 6. Conclusions

This study addresses a central question in the post-pandemic health policy debate: whether variation in hospital performance is primarily driven by exogenous shocks or by structural financing architectures that shape institutional behavior over time. Focusing on psychiatric hospitals within a unified national health insurance system, the analysis disentangles temporal effects from structural configurations in explaining case-mix complexity.

The empirical evidence consistently indicates that psychiatric hospitals do not operate as homogeneous entities reacting uniformly to crisis conditions. Instead, they form distinct financing–organization regimes defined by specific combinations of revenue composition, expenditure allocation, workforce structure, and operational pressure. These regimes are not temporally contingent: although descriptive tests suggest compositional stability, more stringent validation (*ARI* and centroid analysis) shows that structural persistence coexists with moderate reconfiguration across periods. This implies that regimes are not “pandemic-made,” but neither are they fully invariant.

Within the multivariate framework, structural financing characteristics emerge as the primary drivers of case-mix complexity. In contrast, pandemic timing does not retain robust explanatory power once these structural factors are controlled for. This finding supports a structurally grounded interpretation of hospital behavior, in which financing architecture exerts a more durable influence than temporary crisis-related disturbances.

This study contributes to the literature by advancing a typology-based analytical framework that integrates multivariate clustering with outcome modeling. Applied to a longitudinal administrative dataset from a Central and Eastern European context, this approach provides novel empirical evidence in a field largely dominated by studies from Western European and OECD systems. The results highlight that even within a formally unified financing system, institutional heterogeneity remains structured and analytically significant.

At the same time, the relatively low explanatory power of the regression models suggests that case-mix complexity is influenced by additional factors not captured in the current specification, including clinical pathways, referral mechanisms, and unobserved organizational practices. Rather than undermining the results, this limitation reinforces the interpretation that structural financing variables represent only one dimension of a more complex institutional process.

Several limitations should be acknowledged. The use of aggregated administrative data constrains the analysis to institutional-level relationships and does not allow for patient-level or quality-of-care evaluation. The absence of dynamic specifications limits the ability to capture persistence in case-mix evolution, while spatial interactions between hospitals are not explicitly modeled. Future research should extend the analysis using dynamic panel models, spatial econometric approaches, and micro-level clinical data, as well as comparative cross-country designs.

From a policy perspective, the findings indicate that structural incentive design represents a more effective lever than short-term crisis interventions. Financing architecture—through revenue composition, expenditure allocation, and contractual design—shapes hospital behavior in a persistent manner. Consequently, reform efforts should focus less on episodic adjustments and more on the underlying configuration of incentives that governs institutional performance.

Overall, the analysis suggests that psychiatric hospital case-mix complexity is structurally embedded within differentiated financing regimes that persist across temporal disruptions, while allowing for adaptive reconfiguration. By foregrounding financing architecture as a central explanatory dimension, this study contributes to a more nuanced understanding of health system resilience, emphasizing the interplay between structural continuity and institutional adaptation.

## Figures and Tables

**Table 1:** Variables included in empirical analysis.

Variables	Symbol/Formula	Definition
A. Outcome Variables	*CMI*	Case-Mix Index (DRG-based case complexity indicator)
	TCP	Total Cost per Patient
	CASEs	Number of confirmed cases (annual volume)
B. Financing Structure Variables	SH_CNAS	Share of revenues from health insurance contracts in total revenues
	SH_GRANTS	Share of subsidies in total revenues
	SH_VCUR	Share of current revenues in total revenues
	SH_CHELT_CUR	Share of current expenditures in total expenditures
C. Expenditure Structure Variables	SH_PERS	Share of personnel expenditures in total expenditures
	SH_GDS	Share of goods and services expenditures in total expenditures
	SH_CAPEX	Share of capital expenditures in total expenditures
D. Workforce Structure Variables	SH_PSYCH	Share of psychiatrists in total medical staff
	SH_MED	Share of medium-level healthcare staff in total staff
	SH_AUX	Share of auxiliary staff in total staff
E. Operational Pressure Indicators	CASES_PER_BED	CASEs divided by number of beds
	CASES_PER_MED	CASEs divided by number of physicians
F. Temporal Indicators	PAN	Dummy variable = 1 for 2020–2021; 0 otherwise
	POST	Dummy variable = 1 for 2022–2024; 0 otherwise
	2019	Baseline year

**Table 2:** Cluster number of cases.

Cluster		Frequency	Percent	Valid Percent	Cumulative Percent
Valid	1	30	4.0	4.0	4.0
	2	430	57.2	57.2	61.2
	3	292	38.8	38.8	100.0
	Total	752	100.0	100.0	

**Table 3:** Final cluster membership frequencies (three-regime solution).

Cluster ^a^	1	2	3
**Zscore(SH_CNAS)**	−0.23915	−0.03232	0.07216
**Zscore(SH_GRANTS)**	−0.36510	−0.23126	0.37806
**Zscore(SH_VCUR)**	0.16472	0.27352	−0.41972
**Zscore(SH_PERS)**	0.29574	−0.48464	0.68329
**Zscore(SH_GDS)**	−0.10655	0.44195	−0.63987
**Zscore(SH_CAPEX)**	−0.18813	0.21607	−0.29886
**Zscore(SH_PSYCH)**	−0.09727	0.48627	−0.70609
**Zscore(SH_MED)**	−1.27431	0.50894	−0.61854
**Zscore(SH_AUX)**	0.42306	−0.58698	0.82092
**Zscore(CASES_PER_BED)**	3.80503	−0.22529	−0.05917
**Zscore(CASES_PER_MED)**	3.58383	−0.40575	0.22930

^a^ Convergence achieved due to no or small change in cluster centers. The maximum absolute coordinate change for any center is 0.000. The current iteration is 16. The minimum distance between initial centers is 12.652.

**Table 4:** Cross-classification between hierarchical and K-means clustering (k = 3).

Hierarchical (Ward)\K-Means	Cluster 1	Cluster 2	Cluster 3	Total
**Cluster 1**	0	237	0	237
**Cluster 2**	2	192	157	351
**Cluster 3**	28	1	135	164
**Total**	30	430	292	752

**Table 5:** Chi-Square Tests.

Test	Value	df	Asymptotic Significance (2-Sided)
Pearson Chi-Square	1.819 ^a^	2	0.403
Likelihood Ratio	1.839	2	0.399
Linear-by-Linear Association	1.812	1	0.178
N of Valid Cases	752		

^a^ 0 cells (0%) have an expected count less than 5. The minimum expected count is 5.51.

**Table 6:** Contingency matrix between pre-pandemic and post-pandemic cluster assignments.

Cluster PRE/POST	POST 1	POST 2	POST 3	Total
PRE 1	0	2	28	30
PRE 2	237	193	0	430
PRE 3	0	157	135	292
Total	237	351	163	752

**Table 7:** Means and standard deviations (SD) of *CMI* and TCP by cluster and by PAN/POST (MEANS output).

Grouping Structure	N	*CMI* Mean	*CMI* SD	TCP Mean	TCP SD
**By Cluster**					
Cluster 1	30	1.607	0.160	1528.367	114.410
Cluster 2	430	1.570	0.248	1661.684	184.414
Cluster 3	292	1.483	0.276	1590.795	177.186
**By Pandemic Period (PAN)**					
Pre-pandemic (PAN = 0)	614	1.512	0.258	1584.921	177.304
Pandemic (PAN = 1)	138	1.548	0.241	1641.382	186.115
**By Post-Pandemic Period (POST)**					
Pre-post period (POST = 0)	305	1.490	0.247	1532.639	168.921
Post-pandemic (POST = 1)	447	1.570	0.252	1694.479	182.337

**Table 8:** UNIANOVA results for *CMI*: main effects (CLUSTER_FIN, PAN) and interaction (CLUSTER_FIN × PAN), including partial eta squared.

Source	Type III Sum of Squares	df	Mean Square	F	Sig.	Partial Eta Squared
Corrected Model	2.175 ^a^	5	0.435	6.679	0.000	0.043
Intercept	263.806	1	263.806	4051.425	0.000	0.844
CLUSTER_FIN	0.761	2	0.381	5.845	0.003	0.015
PAN	0.023	1	0.023	0.359	0.549	0.000
CLUSTER_FIN × PAN	0.107	2	0.054	0.822	0.440	0.002
Error	48.575	746	0.065			
Total	1828.447	752				
Corrected Total	50.750	751				

^a^ R Squared = 0.043 (Adjusted R Squared = 0.036).

**Table 9:** Levene’s test of homogeneity of variances.

Test (Based on)	F	df1	df2	*p*-Value
Mean	1.097	5	746	0.361

**Table 10:** Panel estimation results and Hausman specification tests (*CMI* and TCP models).

Variables	FE (*CMI*)	RE (*CMI*)	FE (TCP)	RE (TCP)
SH_CNAS	0.476	0.200	150,591.427	135,676.813 *
	(1.441)	(1.035)	(107,804.408)	(80,305.946)
SH_VCUR	−0.239	0.085	−264,589.805 **	−246,724.553 ***
	(1.320)	(1.007)	(98,736.024)	(78,495.253)
SH_PERS	−0.961 *	−1.030 **	−20,837.299	−37,329.586
	(0.513)	(0.468)	(38,410.146)	(36,904.871)
SH_CAPEX	−0.805	−0.852	61,778.854	52,260.173
	(1.045)	(0.985)	(78,184.791)	(77,854.306)
Constant	2.184 ***	2.190 ***	114,325.428 ***	124,076.479 ***
	(0.542)	(0.506)	(40,524.178)	(39,903.111)
Observations	752	752	752	752
R^2^	0.162	0.164	0.307	0.398
**Hausman Test (*CMI*):** χ^2^ = 0.332, *p* = 0.988			**Hausman Test (TCP):** χ^2^ = 1.767, *p* = 0.880	
**Breusch–Pagan LM (*CMI*)** χ^2^ = 15.79, *p* = 0.000			**Breusch–Pagan LM (TCP)** χ^2^ = 22.76, *p* = 0.000	

Notes: Standard errors in parentheses. * *p* < 0.1, ** *p* < 0.05, and *** *p* < 0.01.

**Table 11:** Determinants of Case-Mix Complexity (*CMI*): OLS Estimates.

Model ^a^		Unstandardized Coefficients		Standardized Coefficients	t	Sig.
		B	Std. Error	Beta		
1	(Constant)	2.088	0.435		4.803	0.000
	PAN	−0.025	0.031	−0.038	−0.818	0.413
	POST	0.047	0.026	0.089	1.822	0.069
	SH_CNAS	0.466	0.160	0.143	2.919	0.004
	SH_GRANTS	−0.330	0.367	−0.119	−0.899	0.369
	SH_VCUR	−0.421	0.366	−0.157	−1.151	0.250
	SH_PERS	−0.527	0.268	−0.168	−1.970	0.049
	SH_GDS	−0.076	0.335	−0.020	−0.226	0.821
	SH_CAPEX	−0.051	0.347	−0.008	−0.149	0.882
Model statistics	N	R^2^	Adjusted R^2^	F(8, 743)	*p*	
	752	0.063	0.053	6.218	<0.001	

**Table 12:** Regional OLS Estimates (Robustness Analysis).

Region	R^2^	Significant Structural Variables (*β*, *p*-Value)	Comments
**Region 1**	0.375	SH_CNAS (*β* = 0.512, *p* < 0.001); SH_GRANTS (*β* = −2.147, *p* < 0.001); SH_VCUR (*β* = −2.350, *p* < 0.001); SH_PERS (*β* = −0.494, *p* = 0.014)	Strong structural sensitivity of *CMI* to financing composition.
**Region 2**	0.298	—	No structural or temporal variable reaches statistical significance; *CMI* variation appears comparatively stable and weakly explained by financing composition in this region.
**Region 3**	0.295	SH_CNAS (*β* = 0.491, *p* = 0.002); SH_CAPEX (*β* = 0.536, *p* = 0.024)	Contract revenues and capital intensity increase case-mix complexity.
**Region 4**	0.245	SH_CNAS (*β* = −0.634, *p* < 0.001); SH_GDS (*β* = 0.521, *p* = 0.001); POST (*β* = 0.531, *p* < 0.001)	Structural drivers dominate; limited post-pandemic shift detected.
**Region 5**	0.491	SH_CNAS (*β* = 0.247, *p* = 0.016); SH_GDS (*β* = −0.501, *p* = 0.005)	Financing structure significantly shapes *CMI*.
**Region 6**	0.273	SH_CNAS (*β* = 0.398, *p* = 0.001)	Contract-based revenue share is the primary determinant.
**Region 7**	0.137	SH_GRANTS (*β* = −0.776, *p* = 0.041); SH_CAPEX (*β* = 0.602, *p* = 0.023)	Grants reduce *CMI*; capital intensity increases it.
**Region 8**	0.700	SH_CNAS (*β* = 0.512, *p* < 0.001); SH_GRANTS (*β* = −2.147, *p* < 0.001); SH_VCUR (*β* = −2.350, *p* < 0.001); SH_PERS (*β* = −0.494, *p* = 0.014)	Strong structural sensitivity of *CMI* to financing composition.

## Data Availability

The anonymized analytical dataset used in this study can be made available to interested researchers upon reasonable request to the corresponding author, subject to institutional approval and applicable data-sharing regulations.

## Appendix A

### Appendix A.1. Explained Variables and Formulas Included in Empirical Analysis

**Variables**	**Symbol/Formula**	**Definition**	**Administrative Source**	**Standardized Variables ***
**A.** **Outcome Variables**	*CMI*	Case-Mix Index (DRG-based case complexity indicator)	Clinical reporting	
	TCP	Total Cost per Patient	Financial reporting	
	CASEs	Number of confirmed cases (annual volume)	Clinical reporting	
**B.** **Financing Structure Variables**	SH_CNAS	Share of revenues from health insurance contracts in total revenues	F1	ZSH_CNAS
	${SH\_CNAS}_{it}=\frac{{Revenues\;from\;insurance\;contracts\;(F1|13)}_{it}}{{Total\;revenues\;(F1|1)}_{it}}$			
	SH_GRANTS	Share of subsidies in total revenues	F1	ZSH_GRANTS
	${SH\_GRANTS}_{it}=\frac{S{ubsidies\;(F1|35+F1|40+F1|42+F1|43)}_{it}}{{Total\;revenues\;(F1|1)}_{it}}$			
	SH_VCUR	Share of current revenues in total revenues	F1	ZSH_VCUR
	${SH\_VCUR}_{it}=\frac{{Current\;revenues\;(F1|2)}_{it}}{{Total\;revenues\;(F1|1)}_{it}}$			
	SH_CHELT_CUR	Share of current expenditures in total expenditures	F1	
	${SH\_CHELT\_CUR}_{it}=\frac{{Current\;expenditures\;(F1|57)}_{it}}{{Total\;expenditures\;(F1|56)}_{it}}$			
**C.** **Expenditure Structure Variables**	SH_PERS	Share of personnel expenditures in total expenditures	F1	ZSH_PERS
	${SH\_PERS}_{it}=\frac{{Personnel\;expenditures\;(F1|58)}_{it}}{{Total\;expenditures\;(F1|56)}_{it}}$			
	SH_GDS	Share of goods and services expenditures in total expenditures	F1	ZSH_GDS
	${SH\_GDS}_{it}=\frac{{Goods\;and\;services\;(F1|59)}_{it}}{{Total\;expenditures\;(F1|56)}_{it}}$			
	SH_CAPEX	Share of capital expenditures in total expenditures	F1	ZSH_CAPEX
	${SH\_CAPEX}_{it}=\frac{{Capital\;expenditures\;(F1|65)}_{it}}{{Total\;expenditures\;(F1|56)}_{it}}$			
**D.** **Workforce Structure Variables**	SH_PSYCH	Share of psychiatrists in total medical staff	F2	ZSH_PSYCH
	${SH\_PSYCH}_{it}=\frac{{Number\;of\;physicians\;(F2|6)}_{it}}{{Total\;medical\;staff}_{it}}$			
	SH_MED	Share of medium-level healthcare staff in total staff	F2	ZSH_MED
	${SH\_MED}_{it}=\frac{{Medium-level\;staff\;(F2|11)}_{it}}{{Total\;staff}_{it}}$			
	SH_AUX	Share of auxiliary staff in total staff	F2	ZSH_AUX
	${SH\_AUX}_{it}=\frac{{\mathrm{Auxiliary}\;\mathrm{staff}\;(F2|12)}_{it}}{{Total\;staff}_{it}}$			
**E.** **Operational Pressure Indicators**	CASES_PER_BED	CASEs divided by number of beds	F2	ZCASES_PER_BED
	${CASES\_PER\_BED}_{it}=\frac{CASEs_{it}}{{Number\;of\;beds\;(F2|1)}_{it}}$			
	CASES_PER_MED	CASEs divided by number of physicians	F2	ZCASES_PER_MED
	${CASES\_PER\_MED}_{it}=\frac{CASEs_{it}}{{Number\;of\;physicians\;(F2|6)}_{it}}$			
**F.** **Temporal Indicators**	PAN	Dummy variable = 1 for 2020–2021; 0 otherwise		
	POST	Dummy variable = 1 for 2022–2024; 0 otherwise		
	2019	Baseline year		
	* Used in cluster analysis only.			

### Appendix A.2. Regional Descriptive Statistics for Clustering Variables (N = 752)

**Region**	**Statistics**	***CMI***	**CASEs**	**TCP**	**SH_CNAS**	**SH_GRANTS**	**SH_PERS**	**SH_GDS**	**SH_CAPEX**	**SH_PSYCH**	**SH_MED**	**SH_AUX**	**CASES_PER_BED**	**CASES_PER_MED**	**SH_VCUR**	**SH_CHELT_CUR**
1	Mean	1.394	975.646	1693.400	0.417	0.501	0.755	0.196	0.031	0.115	0.526	0.317	1.765	17.894	0.494	0.973
	SE.Mean	0.019	59.499	17.894	0.006	0.010	0.009	0.007	0.003	0.003	0.004	0.005	0.236	3.309	0.010	0.003
	ST.Dev	0.222	678.391	204.018	0.067	0.110	0.099	0.081	0.036	0.031	0.050	0.058	2.688	37.727	0.109	0.036
2	Mean	1.465	1089.282	1575.769	0.426	0.505	0.768	0.192	0.039	0.104	0.522	0.336	2.506	36.807	0.482	0.965
	SE.Mean	0.020	74.493	17.971	0.008	0.011	0.008	0.005	0.007	0.005	0.005	0.009	0.182	3.358	0.011	0.007
	ST.Dev	0.175	657.901	158.720	0.072	0.100	0.075	0.046	0.064	0.048	0.046	0.081	1.609	29.658	0.095	0.066
3	Mean	1.577	930.742	1561.292	0.450	0.487	0.757	0.202	0.030	0.111	0.528	0.324	2.637	42.493	0.509	0.972
	SE.Mean	0.025	49.331	13.643	0.008	0.007	0.008	0.006	0.004	0.003	0.005	0.006	0.219	5.596	0.007	0.004
	ST.Dev	0.232	465.385	128.712	0.072	0.067	0.076	0.060	0.034	0.025	0.047	0.056	2.070	52.796	0.069	0.034
4	Mean	1.531	956.327	1616.592	0.382	0.476	0.771	0.181	0.037	0.161	0.544	0.260	2.136	11.971	0.519	0.966
	SE.Mean	0.026	94.235	17.516	0.008	0.015	0.012	0.009	0.007	0.003	0.002	0.003	0.223	1.444	0.014	0.007
	ST.Dev	0.181	659.643	122.610	0.058	0.102	0.084	0.063	0.051	0.023	0.016	0.022	1.564	10.108	0.099	0.051
5	Mean	1.526	517.725	1653.044	0.492	0.447	0.700	0.251	0.025	0.114	0.482	0.362	0.903	15.120	0.548	0.978
	SE.Mean	0.023	41.438	26.471	0.010	0.008	0.007	0.005	0.003	0.004	0.007	0.010	0.057	1.692	0.009	0.003
	ST.Dev	0.217	395.296	252.513	0.099	0.079	0.070	0.052	0.026	0.039	0.063	0.094	0.542	16.144	0.081	0.027
6	Mean	1.653	832.119	1645.551	0.427	0.476	0.716	0.232	0.035	0.121	0.534	0.308	1.533	15.537	0.515	0.968
	SE.Mean	0.036	45.331	16.796	0.005	0.008	0.006	0.005	0.003	0.003	0.006	0.005	0.171	2.844	0.008	0.003
	ST.Dev	0.390	492.417	182.453	0.056	0.088	0.068	0.058	0.034	0.030	0.061	0.051	1.856	30.896	0.089	0.034
7	Mean	1.470	667.532	1580.138	0.409	0.484	0.732	0.208	0.043	0.123	0.515	0.332	1.384	14.666	0.494	0.961
	SE.Mean	0.019	37.357	12.349	0.006	0.009	0.008	0.006	0.005	0.004	0.007	0.009	0.056	1.670	0.010	0.005
	ST.Dev	0.184	362.190	119.725	0.057	0.091	0.078	0.055	0.049	0.040	0.064	0.085	0.545	16.189	0.094	0.050
8	Mean	1.683	922.437	1655.650	0.481	0.446	0.687	0.286	0.019	0.126	0.510	0.306	2.240	15.679	0.544	0.983
	SE.Mean	0.017	39.165	17.672	0.009	0.009	0.006	0.006	0.003	0.003	0.005	0.005	0.419	1.761	0.011	0.003
	ST.Dev	0.177	397.480	179.353	0.090	0.088	0.066	0.057	0.033	0.034	0.055	0.048	4.255	17.870	0.107	0.031
Total	Mean	1.538	857.122	1628.839	0.438	0.478	0.733	0.221	0.032	0.120	0.519	0.320	1.846	20.969	0.513	0.971
	SE.Mean	0.009	19.949	6.692	0.003	0.003	0.003	0.003	0.002	0.001	0.002	0.003	0.086	1.170	0.004	0.002
	ST.Dev	0.260	547.064	183.524	0.080	0.093	0.083	0.069	0.041	0.037	0.056	0.070	2.352	32.079	0.097	0.042

### Appendix A.3. Cluster Profiling/External Validation

**Cluster Number of Case**			**Cluster Number of Case × PAN Crosstabulation**			**Cluster Number of Case × POST Crosstabulation**		
			**PAN**		**Total**	**POST**		**Total**
			**0**	**1**		**0**	**1**	
C	1	Count	26	4	30	12	18	30
		% within Cluster Number of Case	86.7%	13.3%	100.0%	40.0%	60.0%	100.0%
	2	Count	356	74	430	165	265	430
		% within Cluster Number of Case	82.8%	17.2%	100.0%	38.4%	61.6%	100.0%
	3	Count	232	60	292	128	164	292
		% within Cluster Number of Case	79.5%	20.5%	100.0%	43.8%	56.2%	100.0%
Total		Count	614	138	752	305	447	752
		% within Cluster Number of Case	81.6%	18.4%	100.0%	40.6%	59.4%	100.0%
